# Analytical evaluation of circulating tumor DNA sequencing assays

**DOI:** 10.1038/s41598-024-54361-w

**Published:** 2024-02-29

**Authors:** Wenjin Li, Xiayu Huang, Rajesh Patel, Erica Schleifman, Shijing Fu, David S. Shames, Jingyu Zhang

**Affiliations:** 1 Oncology Biomarker Development, Roche (China) Holding Ltd, Pudong, Shanghai, China; 2 Oncology Biomarker Development, Genentech, Ltd, South San Francisco, USA

**Keywords:** Cancer, Computational biology and bioinformatics, Biomarkers, Molecular medicine, Oncology

## Abstract

In China, circulating tumor DNA analysis is widely used and numerous assays are available. Systematic evaluation to help users make informed selections is needed. Nine circulating tumor DNA assays, including one benchmark assay, were evaluated using 23 contrived reference samples. There were two sample types (cell-free DNA and plasma samples), three circulating tumor DNA inputs (low, < 20 ng; medium, 20–50 ng; high, > 50 ng), two variant allele frequency ranges (low, 0.1–0.5%; intermediate, 0.5–2.5%), and four variant types (single nucleotide, insertion/deletion, structural, and copy number). Sensitivity, specificity, reproducibility, and all processes from cell-free DNA extraction to bioinformatics analysis were assessed. The test assays were generally comparable or superior to the benchmark assay, demonstrating high analytical sensitivity. Variations in circulating tumor DNA extraction and quantification efficiency, sensitivity, and reproducibility were observed, particularly at lower inputs. These findings will guide circulating tumor DNA assay choice for research and clinical studies, allowing consideration of multiple technical parameters.

## Introduction

Cell-free DNA (cfDNA) is short extracellular DNA fragments that can originate from any cell type located in body fluids^1,2^. Apoptosis and necrosis are thought to be the major mechanisms for release of cfDNA from cells into the peripheral bloodstream^1,3,4^. In the field of oncology, there is much interest in the detection of tumor-derived cfDNA, known as circulating tumor DNA (ctDNA). ctDNA can be distinguished from non-tumor cfDNA via the detection of novel mutations that are not found in normal cells^5,6^; however, there may be challenges related to distinguishing true ctDNA alterations from cfDNA variants related to clonal hematopoiesis^6^. The main benefits of ctDNA diagnostic testing over tissue DNA are the simple requirement of a blood sample rather than a more invasive biopsy, the potential to detect ctDNA shedding from multiple lesions or metastatic sites versus detection only from the biopsied site, and that dynamic changes can be followed over time^7^. Currently, the main application of ctDNA is in precision oncology, where it has the potential to be used in the assessment of prognosis or relapse, detection of minimal residual disease, treatment selection, and monitoring of disease burden and/or resistance^8,9^. In addition, companion diagnostic tests using a next-generation sequencing (NGS) platform with liquid biopsy samples have been approved by the FDA for multiple cancer types and biomarkers^10^.

A major challenge to the clinical application of ctDNA testing is assay sensitivity as, generally, < 1% of the total cfDNA in a patient is tumor-derived^11^. Other challenges include consistency with cfDNA/ctDNA extraction and analysis approaches^12^. In Stetson et al., four NGS gene panel assays were tested using plasma samples to detect mutations in ctDNA^13^. False-negative and false-positive (FP) variants were identified and there was substantial variability in reporting between the four assays^13^. They found that variants with an allele frequency (AF) < 1% had the most discordance, and technical discordance was responsible for 68% of all unique variants identified by the ctDNA assays. Another study comparing the performance of several NGS assays reported that there was substantial variability in sensitivity and specificity, with assay precision being highly dependent on the quantity of DNA input^14^. Accuracy was highest for variants with AFs ≥ 1% and a well-established bioinformatics analysis workflow was found to be important for assay performance^14^. In 2021, an evaluation of the analytical performance of five ctDNA assays, which included evaluations at each stage of the ctDNA sequencing workflow using synthetic DNA spike-in experiments and standardized cell line-derived reference samples, found that there was high sensitivity, precision, and reproducibility for the detection of variant alleles that occurred at a frequency of > 0.5%^15^. Below that threshold, detection was unreliable and varied widely^15^.

We designed and conducted a systematic study to evaluate ctDNA sequencing assays available from nine NGS vendors in China. Assay and vendor names were masked to enable publication of data. Our evaluation included designed reference samples (cfDNA and contrived plasma) to evaluate the technical performance of the platforms for their analytic validity in precision oncology in China. Compared with previously published evaluations, the present study focused on variants with both low (< 0.5%) and intermediate (0.5–2.5%) AFs, as low frequency variants have presented a challenge in previously published studies^15,16^. Study design comparisons with recent ctDNA platform evaluation publications were summarized in Table S1. Most previous studies have limited their evaluations to either cfDNA or plasma samples, but not both, which is why we felt it pertinent to do so. This study evaluated large (> 1 Mb) and small (< 1 Mb) NGS panels and focused on four variant types: single nucleotide variants (SNVs), insertion or deletion variants (InDels), structural variants (SVs, also known as fusion), and copy number variants (CNVs). Vendors with a variety of panel designs and sizes with different limits of detection (LOD) were included in this analysis. These assays are utilized in a variety of ctDNA applications for precision oncology, and the data presented herein are expected to be valuable for informing on the trade-offs of each.

## Results

### Overall study design

**Figure 1:**
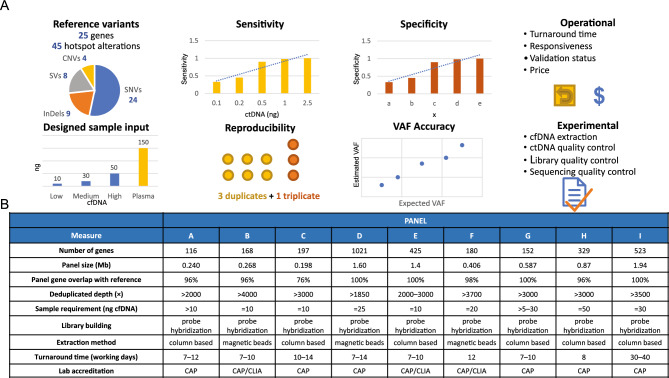
Study design for assessing analytical performance of the ctDNA assays. (**A**) Illustration of the main evaluation aspects and (**B**) basic information for the eight ctDNA assays. *CAP* College of American Pathologists, *cfDNA* cell-free DNA, *CLIA* Clinical Laboratory Improvement Amendments, *CNV* copy number variant, *ctDNA* circulating tumor DNA, *InDel* insertion or deletion variant, *Mb* megabase, *SNV* single nucleotide variant, *SV* structural variant, *VAF* variant allele frequency, *WT* wildtype.

The study design is shown in Fig. 1. In total, 23 reference samples comprising two sample types (diluted cfDNA and synthetic plasma) were employed in this study: 20 cfDNA samples and 3 plasma samples. The cfDNA samples included different dilutions (0% WT, 0.1%, 0.5%, 1%, and 2.5%), cfDNA amounts (10 ng, 30 ng and 50 ng), as well as three pairs of replicates (10 ng at 0.1% and 0.5%; 30 ng at 0.1%). The three plasma samples contained 150 ng of cfDNA with VAFs at 0.2% and were tested in triplicate. The same input amount was provided to each vendor at each VAF concentration. The number of samples and the total amount of cfDNA for each sample by manufacturer’s quantification with cfDNA input categorized as low (< 20 ng), medium (20–50 ng), or high (> 50 ng) after quantification are shown in Table S3. These samples (but not WT) contained 45 hotspot alterations in 25 genes, covering 24 SNVs, 9 InDels, 8 SVs, and 4 CNVs. The aim of this design was to evaluate the sensitivity of each assay at different VAF and cfDNA sample inputs for these four classes of alterations. WT samples were included to evaluate FPs for the specificity analysis and replicates were used to test intra-lab reproducibility.

Panel details for the nine assays selected are shown in Fig. 1; one assay was selected from each vendor using the criteria listed above. Information collected on the overlap of targeted genes and each the LOD of each assay (TableS4 ) informed the design of reference materials, including which variants were included and the dilution gradients created to maximize the robustness of the evaluation. Both large (panel size > 1 Mb; assays D, E, and I) and small NGS panels were included. Only assays that had undergone analytical and clinical validations were chosen, to allow the results to be placed in the context of use in clinical practice. Each assay provider applied their internal standard operating procedures from cfDNA extraction and quantification to downstream bioinformatic pipelines with variant call format files. Summary tables were reported and included cfDNA quantification, mean deduplication depth, and on-target rate. Key experimental details and operational parameters were also evaluated, including sequencer, variant caller, and deduplication method (Fig. 1 and Table S4).

### cfDNA quantification, sequence depth, and on-target rate

A summary of sample quality control (QC) and sequence QC results for each assay are presented in Table 1. Extraction efficiency and sequencing QC were evaluated as they may impact assay sensitivity. We particularly looked at the potential impact from different ctDNA input and variant allele frequency perspective. A comparison of the ctDNA extraction efficiency and quantification for each assay is shown in the supplementary (Fig. S1A,B, TablesS5 , and S6). There is variation among the reported quantification results of both ctNDA and plasma samples regardless of different DNA input and variant allele frequency. And regarding ctDNA extraction from plasma samples, assay F underestimated the cfDNA quantity for the plasma samples, with a mean extraction efficiency of 16%. Since there are variations for final quantification among these vendors even though they were provided with the same amount of samples, it is very important for vendors to have good extraction efficiency and accurate quantification.

**Table 1:** Summary of extraction and sequence QC results.

Assay	cfDNA extraction (min/avg/max)				Sequence QC (min/avg/max)		
	< 20 ng	20–50 ng	> 50 ng	Plasma 150 ng	Mapping percentage	Mean sequence depth	On-target rate
A	7.3/10.4/12.0	23.3/30.7/40.0	52.2/58.4/63.5	107.0/108.7/110.0	0.990/0.998/0.999	7684/13729/31483	0.65/0.70/0.73
B	5.0/9.0/15.0	29.0/36.4/52.0	47.0/53.0/58.0	146.0/150.7/159.0	0.999/0.999/1	1644/6047/10468	0.63/0.68/0.71
C	7.9/12.9/20.4	34.8/40.9/48.0	60.3/65.4/69.3	91.2/104.0/120.0	0.856/0.887/0.908	3256/7555/10551	0.64/0.74/0.78
D (big)	6.1/9.2/12.0	36.1/56.1/107.0	145.0/163.7/187.1	32.4/129.9/223.6	0.995/0.995/0.995	1111/4036/7688	0.59/0.65/0.71
E (big)	11.0/12.9/15.4	28.1/39.6/47.5	39.8/50.1/61.2	145.6/160.5/175.8	0.999/0.999/0.999	1895/2624/3456	0.72/0.72/0.73
F	7.2/10.4/19.6	32.9/36.8/42.4	45.6/52.1/58.0	14.7/24.9/30.5	0.998/0.999/0.999	1515/3417/5022	0.51/0.58/0.64
G	7.5/10.4/14.1	28.4/35.0/50.6	69.1/77.1/90.6	124.3/127.4/131.0	0.993/0.995/0.997	7110/14898/36100	0.54/0.77/0.87
H	5.9/5.8/11.8	23.8/28.1/34.8	41.8/53.5/66.6	108.3/121.9/133.8	0.998/0.999/0.999	2056/4946/7026	0.69/0.71/0.72
I (big)	5.4/6.7/7.6	17.6/18.9/20.3	29.3/32.2/35.0	77.3/83.0/90.2	NA	NA	NA

Assay	Panel sensitivity					Specificity (# FP; Definition 2)	Expected vs. Observed VAF correlation
	0.1%	0.2%	0.5%	1.0%	2.5%	0.0%	< 20 ng/20–50 ng/> 50 ng
A	0.28	0.38	0.76	0.82	0.84	0	0.86/0.81/0.93
B	0.64	0.95	0.90	0.95	0.95	0	0.91/0.96/0.94
C	0.33	0.55	0.68	0.75	0.77	0	0.82/0.88/0.86
D (big)	0.47	0.91	0.96	0.96	0.98	0	0.7/0.91/0.9
E (big)	0.18	0.41	0.68	0.80	0.89	0	0.82/0.81/0.81
F	0.17	0.56	0.73	0.72	0.81	1	0.77/0.82/0.86
G	0.68	0.98	0.94	0.98	0.98	0	0.83/0.88/0.87
H	0.30	0.60	0.81	0.85	0.95	0	0.86/0.91/0.92
I (big)	0.18	0.79	0.55	0.78	0.92	0	0.53/0.87/0.89

*ctDNA* circulating tumor DNA, *FP* false positive, *NA* not available, *QC* quality control, *VAF* variant allele frequency.

Big denotes panel size > 1 Mb.

A comparison of sequencing depth (deduplicated mean depth comparison) is shown in Fig. S1C and Table S7. Sequencing depth varied widely among the assays. Assays A and G had a much higher overall sequence depth (> 10,000×) than the other assays, while assays D, E, and F had a relatively low sequencing depth (< 5000×), with D and E having a larger panel size. The effect of sample input on sequence depth was evaluated. Sequencing depth varied with different cfDNA inputs within the assay. At an input of > 20 ng of cfDNA, all assays reached their expected depth. All samples reached the minimum coverage requirement for analysis and were therefore included in the evaluation. In addition, assays E and F had less variability in deduplicated mean depth compared with the other assays. As expected, a lower cfDNA input tended to have a lower deduplicated mean depth. Adequate sample input is important to ensure high sequence depth in order to detect variants especially with low mutation frequency.

All assays reached an on-target rate of ≥ 50%, which is considered acceptable by vendors for most assays (Fig. S1D and Table S7). Similar to what was observed for sequencing depth, samples with low input tended to have a lower on-target rate. Assay G had higher variability in the on-target rate compared with the other assays, while assays C, E, and H had the lowest on-target rate heterogeneity.

### Overall panel sensitivity and impact factors

**Figure 2:**
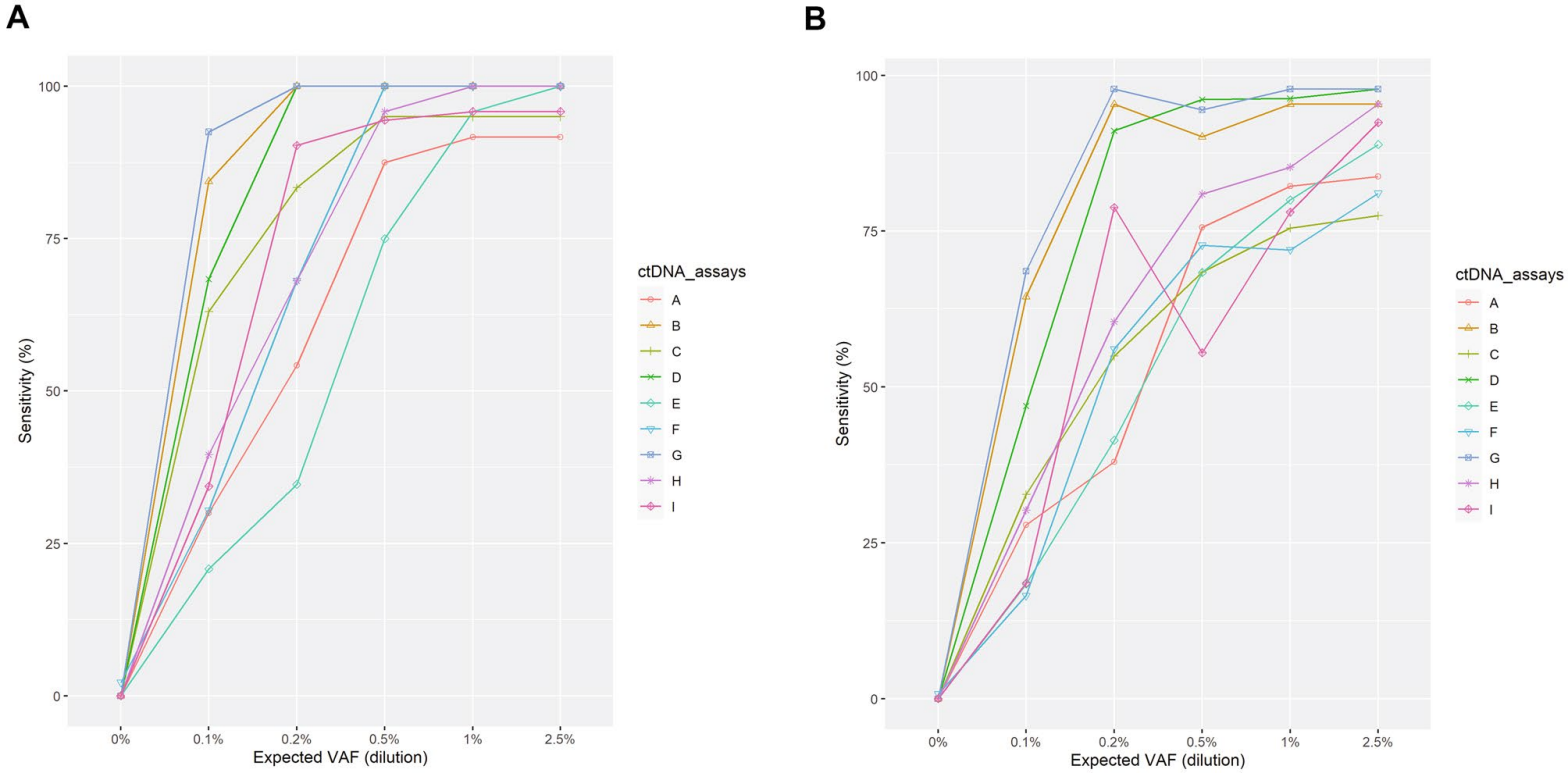
Comparison of assay sensitivity. (**A**) SNV detection sensitivity at LOD and > 20 ng sample input and (**B**) detection sensitivity for all variant types. *LOD* limit of detection, *SNV* single nucleotide variant, *VAF* variant allele frequency.

The sensitivity of each assay was first evaluated by assessing the sensitivity of SNV detection at the required sample input, which was generally > 20 ng (TableS8). All assays except A and E reached a sensitivity of approximately 0.95 for SNV detection at VAF 0.5% (Fig. 2A). The overall sensitivity including all four variant types at different inputs was calculated using on-target known variants in the relevant panel as a reference (Fig. 2B and Table S9). Assays B, D, and G overall had higher sensitivity than the other six assays (including the benchmark assay I) and performed comparably. For all assays, known on-target variants were detected with higher sensitivity in both intermediate VAF (0.5–2.5%) ctDNA samples and synthetic plasma samples when compared with low VAF (0.1%) ctDNA samples. There was a substantial increase in sensitivity for ctDNA samples from VAF 0.1–0.5% for all assays. The increase in sensitivity from VAF 0.5–2.5% was minimal for assays B, D, and G; however, these assays showed high sensitivity (0.90–0.98) at intermediate VAFs (0.5–2.5%) compared with the benchmark assay (0.55–0.92). For all assays, the sensitivity was drastically reduced at VAF 0.1% and ranged from 0.00 to 0.69. Regarding the plasma samples, at VAF 0.2%, three assays (B, D, and G) showed high sensitivity (0.91–0.98), which was better than the benchmark assay (0.79). Some assays had higher or comparable sensitivity in the plasma samples with 0.2% VAF relative to VAF 0.5% cfDNA samples (B, G, and I).

**Figure 3:**
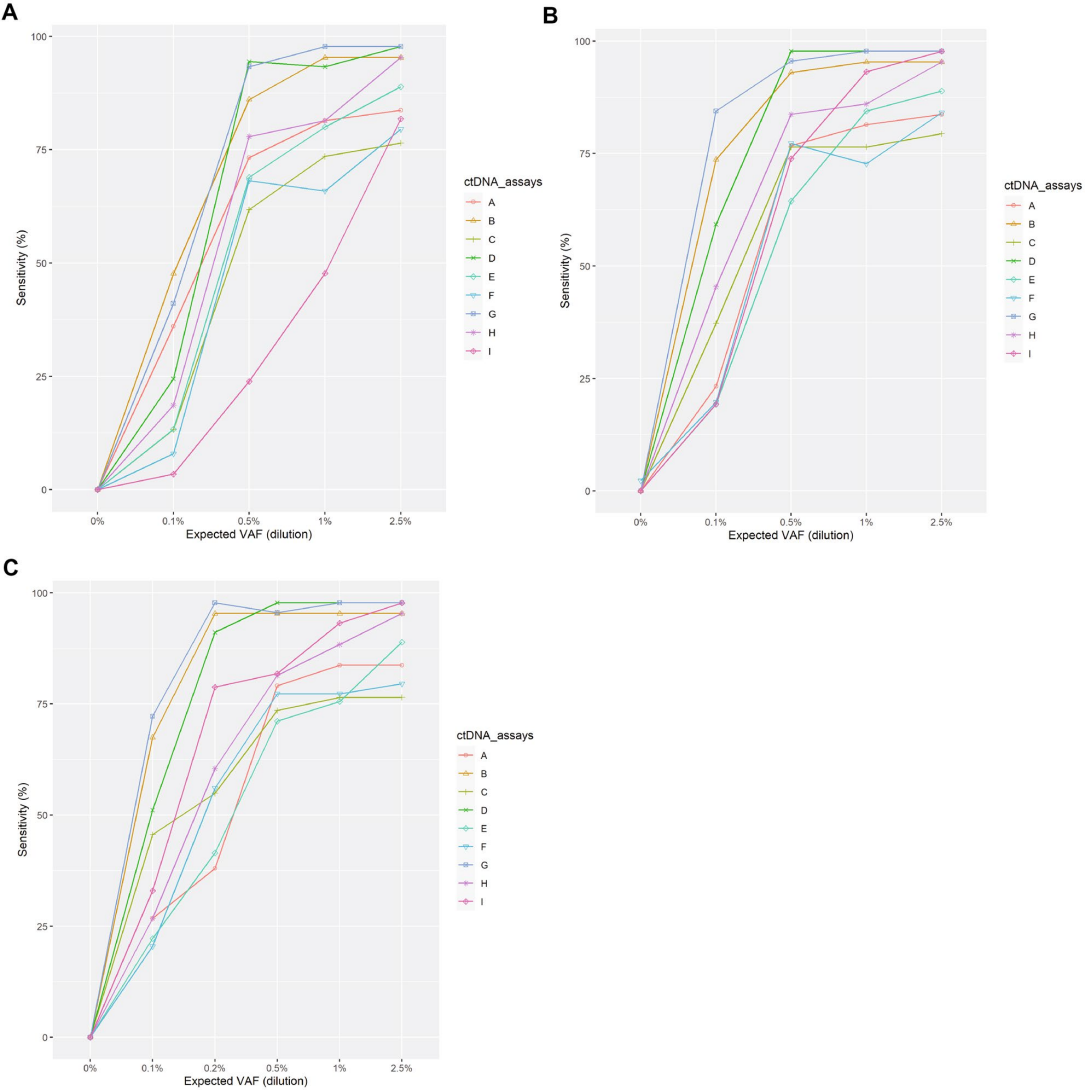
Sensitivity comparison according to cell-free DNA input. (**A**) < 20 ng, (**B**) 20–50 ng, and (**C**) > 50 ng. *VAF* variant allele frequency.

Sensitivity comparisons according to different cfDNA inputs (< 20 ng, 20–50 ng, and > 50 ng) are shown in Fig. 3, Tables S10–S12). For all cfDNA inputs, the trend for sensitivity was similar. For all assays, the sensitivity was more robust with different cfDNA inputs at higher VAFs. There was a substantial increase in sensitivity for most assays, particularly at VAF 0.1%, when cfDNA input changed from low (Fig. 3A) to medium (Fig. 3B). For all assays, there was minimal improvement in sensitivity when cfDNA input changed from medium (Fig. 3B) to high (Fig. 3C). With the medium cfDNA input of 20–50 ng, five assays had a high sensitivity (0.86–0.98) at VAF 1% and a sensitivity above 0.95 at VAF 2.5%.

For the sensitivity comparison according to panel size, three assays (D, E, and I) with a panel size > 1 Mb and six assays (A, B, C, F, G, and H) with a panel size < 1 Mb were evaluated. Among the three > 1 Mb panels, assay D performed best and had comparable performance with the top < 1 Mb assays (B and G). Assay E had the lowest sequence depth but showed comparable performance with the benchmark assay I for cfDNA samples.

### Sensitivity of different variant types

**Figure 4:**
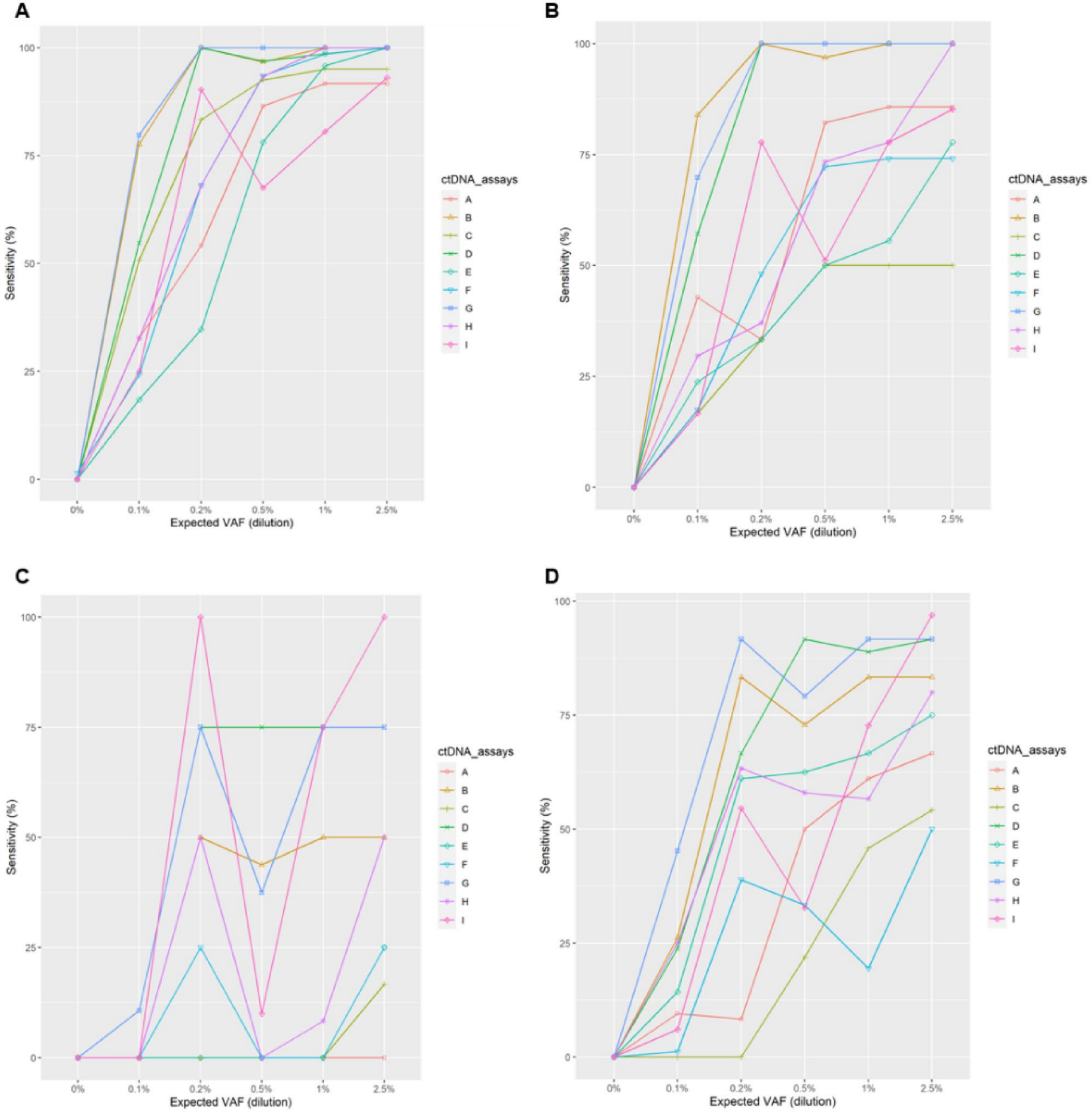
Sensitivity comparison according to variant type. (**A**) SNVs, (**B**) InDels, (**C**) CNVs, and (**D**) SVs using known on-target variants as references. *CNV* copy number variant, *InDel* insertion or deletion variant, *SNV* single nucleotide variant, *SV* structural variant, *VAF* variant allele frequency.

For the cfDNA samples, the sensitivity comparison for on-target SNVs, using on-target known variants in the panel as reference, showed a similar pattern as the overall sensitivity and is shown in Fig. 4A and Table S13. Assays B, D, and G showed overall higher sensitivity than the other six assays; however, the difference was not as notable as that for on-target known variants. Additionally, most of the assays had better or comparable sensitivity versus the benchmark assay at intermediate VAFs. All assays had high sensitivity above 0.9 at VAF 2.5%, and all assays except I had a high sensitivity (0.92–1) at VAF 1%. At VAF 0.5%, six of the assays had a high sensitivity (0.92–1). With VAF 0.1%, sensitivity for all assays decreased notably; however, two of the assays had a sensitivity above 0.75 (B and G). Using panel variants as reference, sensitivity comparisons for on-target InDel variants are shown in Fig. 4B. Assays B, D and G had superior performance compared with the other assays; sensitivity increased substantially from VAF 0.1–0.5%. There was little increase in sensitivity for most assays from VAF 0.5–2.5%. All assays except C, E, and F had a sensitivity > 0.85 at VAF 2.5%. Five assays had better sensitivity than the benchmark with VAF 2.5%. Assays A, B, D, and G had relatively higher sensitivity at VAF 1% (0.86–1) and VAF 0.5% (0.82–1). For all assays, the sensitivity was drastically reduced at VAF 0.1%; only assay B had a sensitivity > 0.75. Sensitivity comparisons for on-target CNVs when using panel variants as reference are shown in Fig. 4C. For cfDNA, all assays except the benchmark assay performed poorly in detecting CNVs, even at VAF 2.5%. At VAF 2.5%, the benchmark assay was the only one with all four CNVs detected; assays D and G detected three (at both VAF 2.5% and 1%). For the plasma samples, the benchmark assay was also the only one with all four CNVs detected at VAF 0.2%.

Sensitivity comparisons for on-target SVs when using panel variants as a reference were also evaluated among the different assays (Fig. 4D). Five assays (B, D, E, G, and H) had better overall performance than the other assays. Sensitivity increased substantially from VAF 0.1–0.5% for all assays and a minimal increase was observed for assays B, D, E, G, and H from VAF 0.5–2.5%. Most assays had better sensitivity than the benchmark assay at different dilutions. At VAF 2.5%, all assays except C and F had a sensitivity > 0.95; at VAF 1%, five assays (A, B, D, E, and G) had high sensitivity (0.92–1); and at VAF 0.5%, four assays (B, D, E, and G) had high sensitivity (0.88–1). Only one assay (G) had a sensitivity > 0.6 at VAF 0.1%. For the plasma samples, there were three assays with a high sensitivity (0.92–1) at VAF 0.2%; this was higher than the benchmark (0.29). Sensitivity results for on-target SNV, InDel, CNV, and SV when using the list of detected variants at VAF 2.5% as a reference were similar to those obtained when using panel variants as the reference (Fig. S2 and Fig. S3A–D, respectively).

### Specificity assessment

A FP was defined as the number of the designed variants detected in WT cfDNA samples. Specificity analysis showed that an FP was only detected in assay F (TP53p.R175H); these results are summarized in Table 1.

### Reproducibility evaluation

**Figure 5:**
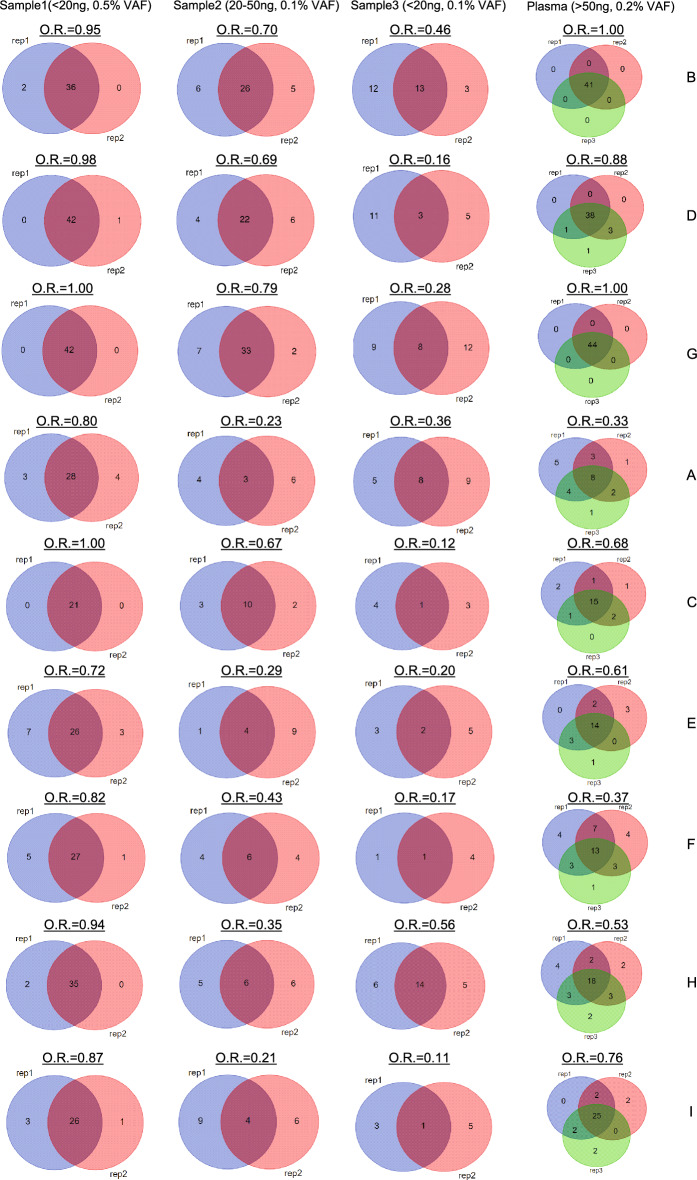
Reproducibility of assays. cfDNA samples with different combinations of cfDNA input and VAF were assayed in duplicate and plasma samples (cfDNA > 50 ng, VAF 0.2%) were assayed in triplicate.

The design of the study to evaluate panel reproducibility included three cfDNA samples (sample 1, sample 2, and sample 3), which were tested in duplicate, and one plasma sample, which was tested in triplicate. For all assays, reproducibility was affected by both the quantity of cfDNA input and VAF (Fig. 5). All assays had high reproducibility with sample 1 (cfDNA < 20 ng, VAF 0.5%) with six showing a reproducibility > 0.85. For sample 2 (cfDNA 20–50 ng, VAF 0.1%), assays B, C, D, and G had higher reproducibility (0.67–0.79) relative to the other assays (0.21–0.42). All assays had poor reproducibility with sample 3 (cfDNA < 20 ng, VAF 0.1%), ranging from 0.12 to 0.56. Although the cfDNA input was the same for samples 1 and 3, the higher VAF in sample 1 greatly improved the reproducibility compared with sample 3. For the plasma samples, assays B, D, and G had the highest reproducibility (0.88–1).

### VAF accuracy

**Figure 6:**
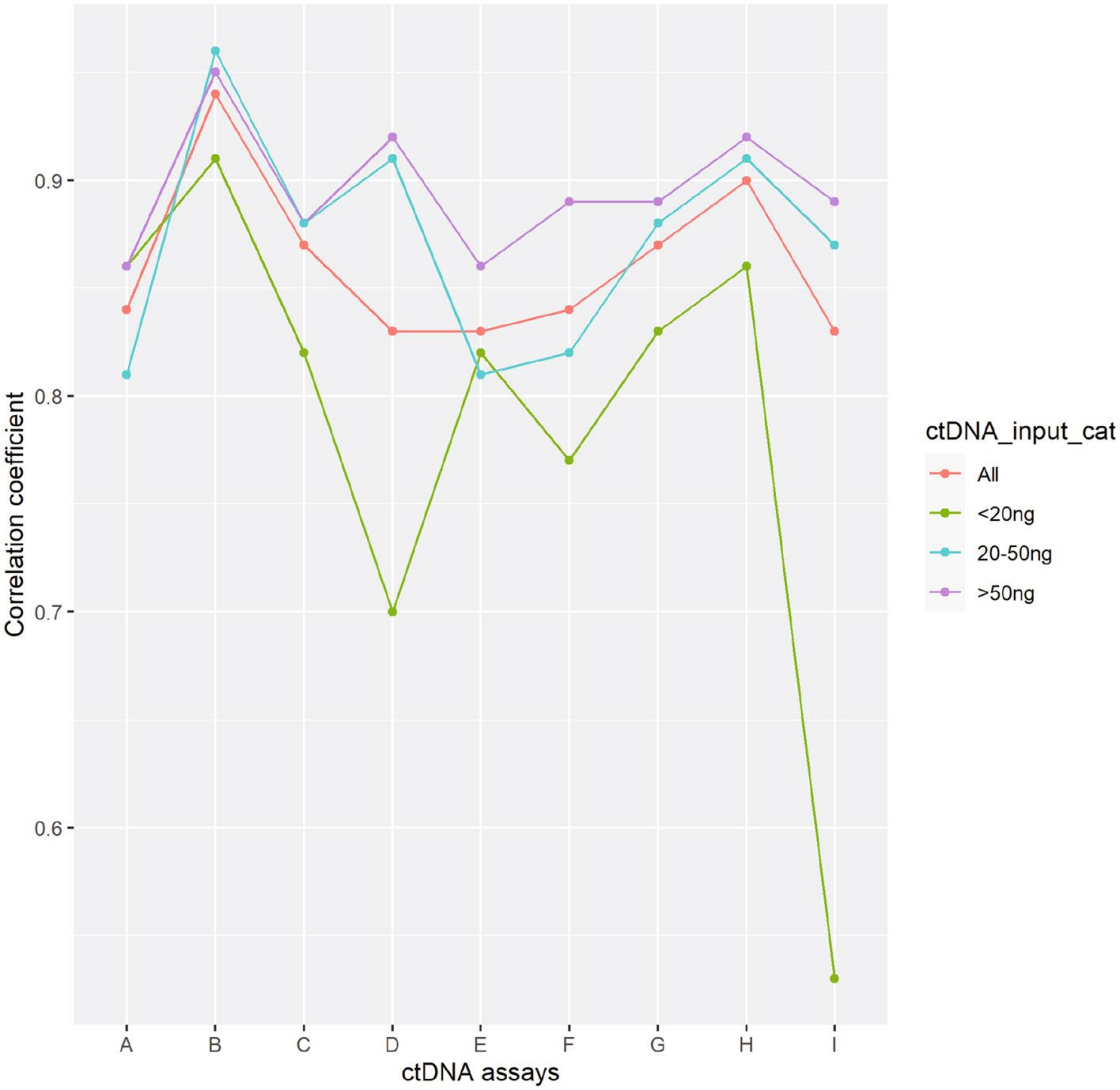
Comparison of the correlation between the expected and observed VAF according to cfDNA concentration. Pearson’s correlation of the observed allele frequency and the known allele frequency were calculated for each assay and compared among the different assays according to cfDNA input. cfDNA, cell-free DNA.

The correlation coefficient between the expected and observed VAF is plotted in Fig. 6 and Table S14. For most assays, the correlation coefficient was high (> 0.80) at different ctDNA inputs; assays D and I were exceptions as the correlation coefficient improved substantially with medium to high cfDNA input. Assay B tended to have the highest correlation coefficient of the assays at each ctDNA input. With a low cfDNA input, assays A, B, and H had higher correlation coefficients than the others, while at an input of > 20 ng, assays B, D, and H had higher correlation coefficients than the others. Overall, the detection accuracy decreased with lower sample input.

## Discussion

ctDNA assays are making a significant impact in drug development and patient care. Given the many emerging use cases in the clinical setting, covering a variety of applications, it is critical to consider technical and analytical characteristics when choosing an appropriate ctDNA sequencing assay. Along with the considerable global interest in developing ctDNA technologies, ctDNA sequencing assays are rapidly evolving in China. However, a systematic understanding and evaluation of the overall readiness of these new assays, compared with commercially available and globally accessible assays, is lacking. To this end, this study provides the first large-scale and wide-range assessment of nine ctDNA assays available in China, using customized SeraCare contrived samples.

To maximize the effectiveness of this evaluation using reference samples, the present study selected panels with the largest overlap with the variants contained in the reference samples. Additionally, panels with better validation for applications in real-world clinical practice were preferentially selected. The present evaluation enabled the assessment of sensitivity, specificity, reproducibility, and accuracy through the design and use of different variant types, a range of VAFs (0.1% [low] to 2.5% [medium]), different cfDNA inputs (< 20 ng, 20–50 ng, and > 50 ng), as well as replicate samples and different sample types. Using designed reference samples, the present study showed that ctDNA sequencing assays from leading NGS vendors in China were generally comparable to or better than the benchmark assay, demonstrating a high potential for clinical utility. Performance differences may have implications for assay utility at different testing points along the patient journey. The results of the present study provide a comprehensive overview of the NGS-based ctDNA assay landscape in China, which is expected to help inform clinicians and researchers on the selection and application of ctDNA technology in precision oncology.

Multiple studies have shown that low-frequency mutations commonly occur in early-stage cancer^17,18^ and in post-surgical minimal residual disease with VAF < 0.1%^18,19^. In addition, we observed that around 25% of patients had VAF < 0.5% for specific hotspot SNVs (unpublished data). This calls for more sensitive assays to detect mutations at low VAF, especially for early-stage cancers. In the present study, assays B, D, and G had the greatest sensitivity regardless of variant type, variant VAF, ctDNA input, or sample type. SNVs with VAF ≥ 0.5% (0.5%, 1.0%, and 2.5%) were detected with high sensitivity using seven of the nine participating assays. However, the detection of SNVs below VAF 0.5% varied widely among the assays, with five having sensitivity > 0.8 at 0.2%, and assays B and G having the greatest sensitivity (> 0.75) at 0.1%. It is noted that some samples had cfDNA concentrations that reached the lower limit for some of the assays evaluated (Table S4), which may have resulted in an underestimation of assay performance. This finding is in line with recently published results of ctDNA platform evaluations^15,16^ and is, in general, similar to the publicly available information from several ctDNA sequencing providers that have been widely used in global clinical practice^20,21^.

Non-SNVs, such as Indels, SVs, and CNVs, are also actionable cancer biomarkers^22–26^, and as such, there is a need for effective liquid biopsy assays to detect these variant types. For non-SNV types, we observed that assay performance is generally worse than that for SNVs and varies dramatically among different assays. Among the three non-SNV types tested in this study, InDels were detected with high sensitivity (> 90%) by three, three, and four assays at VAF 0.5%, 1%, and 2.5%, respectively. They were detected at high sensitivity by three assays at VAF 0.2% and two assays at VAF 0.1% with sensitivity ≥ 0.7. SVs were detected with high sensitivity (> 0.90) for four, five, and seven participating assays at VAF 0.5%, 1.0%, and 2.5%, respectively. SVs were detected by only three assays at VAF 0.2% (sensitivity > 0.9) and by only one assay at VAF 0.1% (sensitivity approximately 0.60); this is generally lower than what has been reported for the Guardant360 assay^15^. For CNVs, our evaluation focused more on VAF 2.5%, as the copy number is diluted and may reach undetectable levels at lower VAFs in the other cfDNA samples. As a result, all assays except the benchmark assay performed poorly in detecting CNVs. One possible influencing factor could be the covered target region of the gene among the different assays^27^. Additionally, it was reported that the sensitivity of ctDNA CNV detection increases with high ctDNA fraction, high copy number change (≥ 4/copy), and high sequence depth^27^. The low CNV amplification design (average of 3) and limited sequence depth in most of the assays may have limited the analyses of CNV detection in our study. The above data suggest that improved performance is needed for most assays for the detection of non-SNVs.

In addition to VAFs and variant types, sample input was an influencing factor on detection sensitivity. Several cfDNA samples with low input at various VAFs were incorporated in the present study, and most assays were able to extract ~ 10 ng ctDNA from those samples. We found that low ctDNA input tended to have lower sequence depth (< 3000×) for most assays. As a result, the overall sensitivity for samples with low input was worse than that from higher input; the differences were more prominent at low VAF. Similarly, some assays had higher sensitivity with VAF 0.2% (plasma samples) than with VAF 0.5% (cfDNA samples), which may have been because plasma samples had an adequate amount of cfDNA input. When evaluating the impact of sequence depth and panel size on sensitivity, neither seemed to play a significant role independently. Assays A and G had an overall higher sequence depth than the other assays; however, assay A did not perform as well as assay G. Three assays (D, E, and I) with large panels (panel size > 1 Mb) were included in the study with assay D among the top performing assays.

To avoid over treatment in the clinic, a low FP rate is an important feature of assays to be used in clinical settings. In the present study, specificity was high for all assays; eight of the nine assays did not detect any FPs using the detection of any variant in the WT ctDNA sample as the criteria. Therefore, compared with sensitivity, specificity is less of a differentiating factor for assay performance; this is consistent with a similar study that was recently published that reported low FP rates^28^. It should be noted that the specificity estimation focused on the FP results when defined as the detection of any designed variants in WT cfDNA samples rather than other background noise. This may overestimate the performance of specificity.

This study provides insight on performance related to VAF accuracy. Accuracy on VAF reporting is not strictly required when it comes to the clinical utility of cancer detection and molecular stratification in cases where determining the presence or absence of target variants is sufficient. Nevertheless, VAF precision is critical for therapeutic monitoring and post-treatment surveillance. For example, when a more accurate representation of the true amount of ctDNA was needed, absolute ctDNA levels (as determined by normalizing VAFs by plasma volume), were used to explore the predictive value in some solid tumors^29,30^. Here, accurate VAF calling was found to be the underlying foundation. In hematological malignancies, this was demonstrated as the log-fold change in ctDNA from baseline to on treatment, which was used as a prognostic factor during first-line treatment of patients with diffuse large B-cell lymphoma and relied on the stringent detection of VAF^31^. In the present study, VAF accuracy was higher with increased sample input, emphasizing the importance of sufficient cfDNA to guarantee solid VAF readouts in clinical practice. Additionally, assays B, D, and H demonstrated more accurate VAF than the other assays, with a coefficient > 0.9 when there was medium-to-high cfDNA input where asays B, D, H showed relatively high deduped depth compared with other assays (Fig. S1). At low cfDNA input, it is interesting to be noted that assay A showed a generally good VAF accuray (0.86 in Table S13), which may be partially due to its relatively high sequencing depth at low input (samples marked red of box A in Figure C of Fig. S1). It is consistent with previous findings that fragment depth may affect the detection accuracy^15,20^. These observations suggest that sample input and sequence depth account for the level of VAF accuracy.

Experimental factors of the different assays had some effect on assay performance. The observed fluctuations of reported cfDNA input reflected, to some degree, both the extraction efficiency and quantification procedures, which can play a part in assay performance given that inaccurate sample input estimation may further affect downstream library construction and thus potentially impact the optimized ctDNA assay workflow. The capability of handling different samples was also evaluated by including plasma samples, which exhibited a similar extraction efficiency to cfDNA samples. Additionally, we did not observe a prominent effect on technical performance from on-target rate, sequencer and extraction method, which was likely because all the assays were optimized to be robust and were analytically validated with these variables.

Aside from the technical aspects, operational features should also be taken into consideration when performing assay selection in clinical practice. All the vendors of the assays that were included in the present study were accredited by the College of American Pathologists and all the assays have finished the analytical validations. Turnaround time should also be given special consideration when ctDNA testing is used prospectively, as this allows clinicians to receive information on ctDNA status in time to aid in therapy selection or, in the case of clinical trials, patient selection or stratification. Moreover, the responsiveness across all the vendors are all highly efficient. Additionally, the price is comparable except assay I (data not shown), which offered high price, although the actual quotation may vary depending on sample volume and operational requirements in the clinical setting.

In conclusion, this study evaluated multiple aspects of performance related to ctDNA detection across nine NGS assays available in China using designed reference cfDNA samples. The results of this study provide a framework and guidance for researchers and clinicians when selecting a ctDNA assay for specific applications within precision oncology. The cfDNA extraction efficiency, sequence depth, on-target rate, mutation frequency, mutation type, sample input are the main technical factors influencing ctDNA detection performance, which include the sensitivity, specificity and accuracy. How to increase the ctDNA input material and improve the coverage depth are the most critical variables in clinical translation of ctDNA assays. As contrived samples may not reflect the complexity of patient samples, assay performance should be further evaluated and improved in the clinical setting using patient samples. Furthermore, innovative approaches that allow the examination of ctDNA fragment size and ctDNA methylation are expected to play a part in increasing the resolution of ctDNA assays, which need to be further developed and accessed.

## Materials and methods

### cfDNA samples

The cfDNA samples used in this study were obtained from SeraCare Life Sciences (Milford, MA, USA) and consisted of 45 cancer DNA variants (Table S2) spiked into a background of wildtype (WT) genomic DNA (derived from cell line GM 24385, Coriell Institute for Medical Research, Camden, NJ, USA), with variants at a target AF of 2.5 ± 0.75; at least 95% of variants were within the acceptable range. The ctDNA variants included 45 hotspot alterations in 25 genes with 24 SNVs, nine InDels, eight SVs, and four CNVs (3.2 ± 0.5 total copies per CNV). To create the samples, cfDNA was prepared to a length of approximately 170–180 base pairs using a proprietary SeraCare process (https://www.seracare.com/resources-and-education/seraseq-ngs-reference-materials/).

### Plasma samples

For the synthetic plasma samples, 0.2% of the custom cfDNA reference material described above (SeraCare) was added at approximately 30 ng/mL in 5 mL contrived plasma (total extractable cfDNA of approximately 150 ng). The AF target of each variant within this library was a 0.2% (0.14–0.26%) AF mix; at least 95% of variants were within the acceptable range. The CNVs in these plasma samples had a target average total copy number of 3.2 ± 0.5 per CNV as confirmed by droplet digital polymerase chain reaction (PCR).

### Assay selection

This study included nine NGS-based ctDNA assays available in China. Assays were selected based on their panel design, validation status, LOD, and the qualifications of the laboratories performing the testing (e.g., College of American Pathologists/Clinical Laboratory Improvement Amendments accreditation; Fig. 1). Assay I, developed by a US-based company and applied globally, was chosen as the benchmark assay in this study. All the assays applied probe hybridization capture for target sequencing library building. A total of 23 reference samples were tested by all the assays, including 20 cfDNA samples and three plasma samples. Samples were sequenced using sequencing platforms and pipelines at each vendor site.

### Sample preparation

WT cfDNA and 2.5% cfDNA were mixed to generate samples at different variant AFs (VAFs). After verifying these VAFs (SeraCare), the 1.0%, 0.5%, and 0.1% AF mixed samples were produced at 1.75 ng/μL, 1.25 ng/μL, and 0.5 ng/μL by further diluting the 2.5% mutant cfDNA with WT cfDNA according to mass as quantified using the Qubit dsDNA High Sensitivity assay kit (ThermoFisher Scientific, Waltham, MA, USA) with three input amounts designed (10 ng, 30 ng, 50 ng). Each cfDNA mixture was separated into 44 μL aliquots; 4 μL was used for quantification by the vendor and 40 μL was used for cfDNA extraction. The 2.5% mutant cfDNA mix had an average total copy number of 3.2 per CNVs as determined by droplet digital PCR. Samples containing 1.0%, 0.5%, and 0.1% CNVs were generated by blending the 2.5% sample with WT cfDNA according to mass to reach the desired dilution.

### cfDNA extraction and quantification

cfDNA was extracted using a QIAamp circulating nucleic acid kit (Qiagen, Germantown, MD, USA). cfDNA concentrations were verified using the Qubit dsDNA Broad Range assay kit (ThermoFisher Scientific) by SeraCare and cfDNA input was quantified and categorized as low (< 20 ng), medium (20–50 ng), or high (> 50 ng), which was used as input classification for comparison. Digital PCR testing was performed to verify the presence of variants prior to the shearing process for both the 2.5% mix and the plasma samples containing 0.2% reference material. Upon the receipt of plasma samples, participant vendors performed cfDNA extraction, quantification, and all other internal procedures.

### Statistical analyses

All statistical analysis was performed using R Statistical Software version 4.0.3^32^.

### Evaluation of sensitivity

Assay sensitivity was defined as the number of on-target known variants detected in a given sample divided by either the number of on-target known variants in the panel (presented here) or by the maximum on-target known variants detected at VAF 2.5% (presented in the supplement). It was calculated within pre-defined expected VAFs (0.1%, 0.2%, 0.5%, 1% and 2.5%) globally and within specific ctDNA inputs, or within specific variant types.

### Evaluation of specificity

FPs were defined as the number of on-target known variants present in WT samples. FPs were adjusted for the relevant assay panel size.

### Evaluation of reproducibility

When comparing replicates of the same sample within the participating assays, reproducibility was defined as the portion of variants detected in all replicates relative to the total number of variants detected in each of the replicates.

### Evaluation of VAF accuracy

VAF accuracy was evaluated by the correlation of the observed and expected VAFs of all reported true-positive variants. Pearson’s correlation was calculated for each assay both globally and within each cfDNA input category.

### Supplementary Information

Supplementary Legends.

Supplementary Figure S1.

Supplementary Figure S2.

Supplementary Figure S3.

Supplementary Table S1.

Supplementary Table S2.

Supplementary Table S3.

Supplementary Table S4.

Supplementary Table S5.

Supplementary Table S16.

Supplementary Table S7.

Supplementary Table S8.

Supplementary Table S9.

Supplementary Table S10.

Supplementary Table S11.

Supplementary Table S12.

Supplementary Table S13.

Supplementary Table S14.

## Data Availability

All data generated or analysed during this study are included in this published article (and its Supplementary Information files).
