# Supplementary material for: Analytical evaluation of circulating tumor DNA sequencing assays
Source: Sci Rep. 2024 Feb 29;14:4973. doi: 10.1038/s41598-024-54361-w (PMC10904763; doi:10.1038/s41598-024-54361-w)
Supplement: Supplementary file 1 — Supplementary Legends. [file 41598_2024_54361_MOESM1_ESM.docx]

**Supplementary information for**

**Analytical evaluation of circulating tumor DNA sequencing assays**

Short title: Circulating tumor DNA sequencing assay evaluation

Wenjin Li^1,¶^, Xiayu Huang^1,¶^, Rajesh Patel^2,¶^, Erica Schleifman^2^, Shijing Fu^1^, David S. Shames^2,*^, Jingyu Zhang^1,*^

^1^ Oncology Biomarker Development, Roche (China) Holding Ltd, Pudong, Shanghai, China

^2^ Oncology Biomarker Development, Genentech, Ltd, South San Francisco, United States

^¶^ These authors contributed equally to this work

^*^Correspondence:
E-mail: jingyu.zhang@roche.com (JZ) and shames.david@gene.com (DSS)

# Supporting information captions

**Fig. S1 Comparison of (A) cfDNA and plasma extraction and quantification efficiency (observed DNA input), (B) cfDNA and plasma mean extraction efficiency, (C) sequence depth, and (D) on-target rate.** Related to Table 1. Variations of sample extraction and quantification were observed among vendors. Low DNA input will result in low sequence depth and on target rate.

AF, allele frequency; cfDNA, cell-free DNA

**Fig. S2 Comparison of assay sensitivity overall using VAF 2.5% as a reference.** Single nucleotide variants ≥20 ng (A) and overall (B). Related to Table 1. Similar patterns of sensitivity results among different assays were observed using the variants detected at 2.5% VAF as reference compared with the sensitivity results derived from using the overlapped variants in the panel as reference.

VAF, variant allele frequency

**Fig. S3 Sensitivity comparison according to variant type (A) SNVs, (B) InDels, (C) CNVs, and (D) SVs using VAF 2.5% as a reference.** Related to Fig 2. Similar patterns of sensitivity results among different assays with individual variant type were observed using the variants detected at 2.5% VAF as reference compared with the sensitivity results derived from using the overlapped variants in the panel as reference.

CNV, copy number variant; InDel, insertion or deletion variant; SNV, single nucleotide variant; SV, structural variant; VAF, variant allele frequency.

**Table S1. Study design comparisons with recent ctDNA platform evaluation publications.**

**Table S2. Details of the 45 reference variants used in the study.**

CNV, copy number variants; InDel, insertion or deletion variants; SNV, single nucleotide variants; SV, structural variants.

ID, identification.

**Table S3. Number of samples at each dilution according to total amount of cfDNA.**

cfDNA, cell-free DNA.

**Table S4. Key experimental details and operational parameters.**

amp, amplification; CNV, copy number variants; dup, duplication; del, deletion; InDel, insertion or deletion variants; LOD, limits of detection; NA, [mutation type not reported]; SNV, single nucleotide variants; SV, structural variants; UMI, [Unique Molecular Identifier].

**Table S5. cfDNA extraction quantification.** Related to S1A Fig.

cfDNA, cell-free DNA; ctDNA, circulating tumor DNA.

**Table S6. cfDNA Mean extraction efficiency.** Related to S1B Fig.

**Table S7. Deduplicated sequence depth and on-target rate.** Related to S1C Fig and S1D Fig.

cfDNA, cell-free DNA; ctDNA, circulating tumor DNA.

**Table S8. Assay single nucleotide variant sensitivity with sample inputs >20 ng.** Related to Figure 2A and S2 FigA.

ctDNA, circulating tumor DNA; SNV, single nucleotide variant.

**Table S9. Assay overall sensitivity with sample inputs >20 ng.** Related to Figure 2B and S2. FigB

ctDNA, circulating tumor DNA.

**Table S10. Assay overall sensitivity with sample inputs <20 ng.** Related to Figure 3A.

ctDNA, circulating tumor DNA.

**Table S11. Assay overall sensitivity with sample inputs 20–50 ng.** Related to Figure 3B.

ctDNA, circulating tumor DNA.

**Table S12. Assay overall sensitivity with sample inputs >50 ng.** Related to Figure 3C.

ctDNA, circulating tumor DNA.

**Table S13. Assay overall sensitivity with different variant types.** Related to Figure 4A–D and S3 FigA–D.

CNV, copy number variants; ctDNA, circulating tumor DNA; InDel, insertion or deletion variants; SNV, single nucleotide variant; SV, structural variants.

**Table S14. Assay allele frequency correlation at different sample inputs.** Related to Figure 6.

ctDNA, circulating tumor DNA.
