# Supplementary material for: Analytical evaluation of circulating tumor DNA sequencing assays
Source: Sci Rep. 2024 Feb 29;14:4973. doi: 10.1038/s41598-024-54361-w (PMC10904763; doi:10.1038/s41598-024-54361-w)
Supplement: Supplementary file 2 — Supplementary Figure S1. [file 41598_2024_54361_MOESM2_ESM.docx]

**
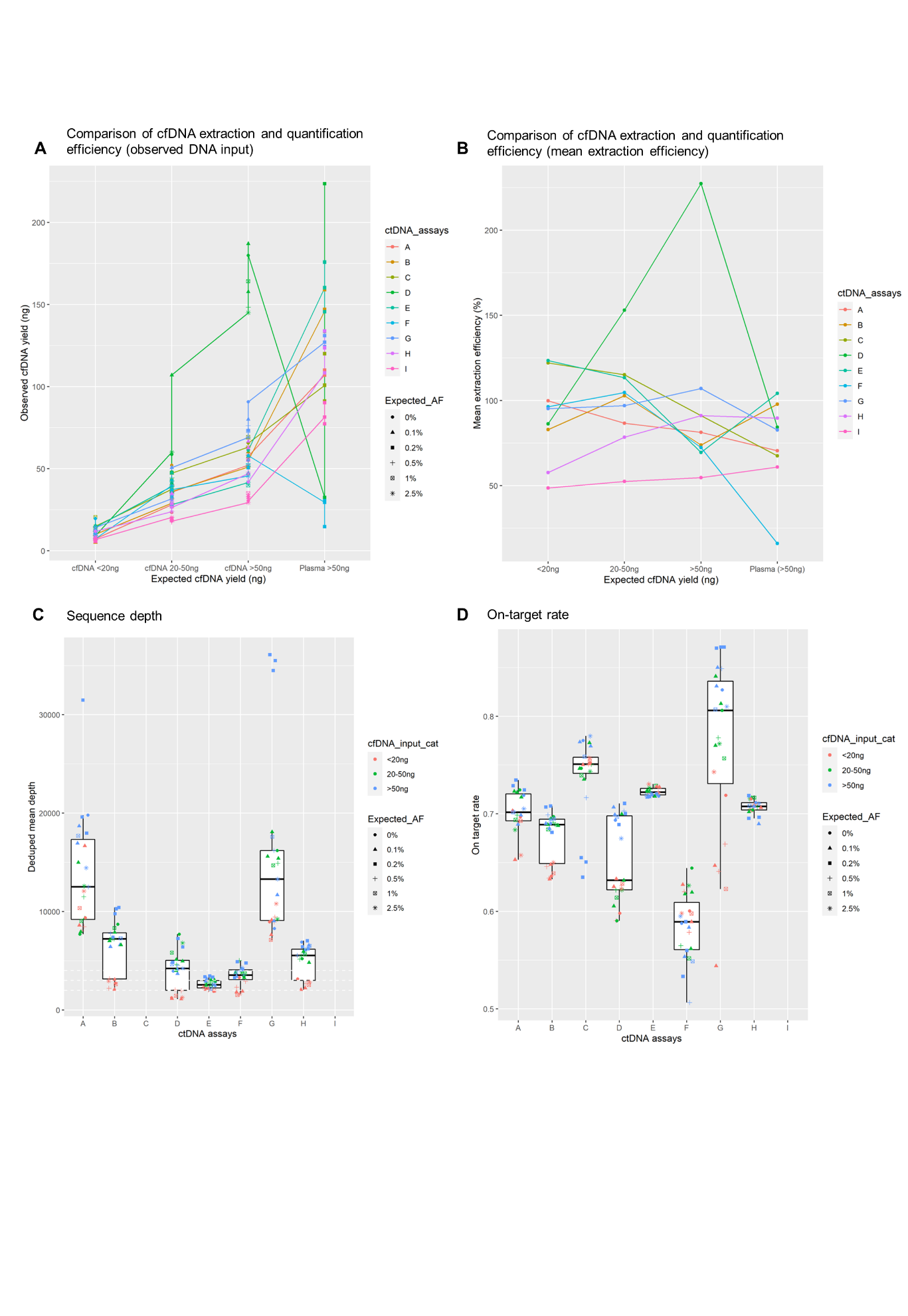
**

**Fig.** **S1 Comparison of (A) cfDNA extraction and quantification efficiency (observed DNA input), (B) cfDNA extraction and quantification efficiency (mean extraction efficiency), (C) sequence depth, and (D) on-target rate.** Related to Table 1. Variations of sample extraction and quantification were observed among vendors. Low DNA input will result in low sequence depth and on target rate.

AF, allele frequency; cfDNA, cell-free DNA
