# Supplementary material for: Analytical evaluation of circulating tumor DNA sequencing assays
Source: Sci Rep. 2024 Feb 29;14:4973. doi: 10.1038/s41598-024-54361-w (PMC10904763; doi:10.1038/s41598-024-54361-w)
Supplement: Supplementary file 3 — Supplementary Figure S2. [file 41598_2024_54361_MOESM3_ESM.docx]

**
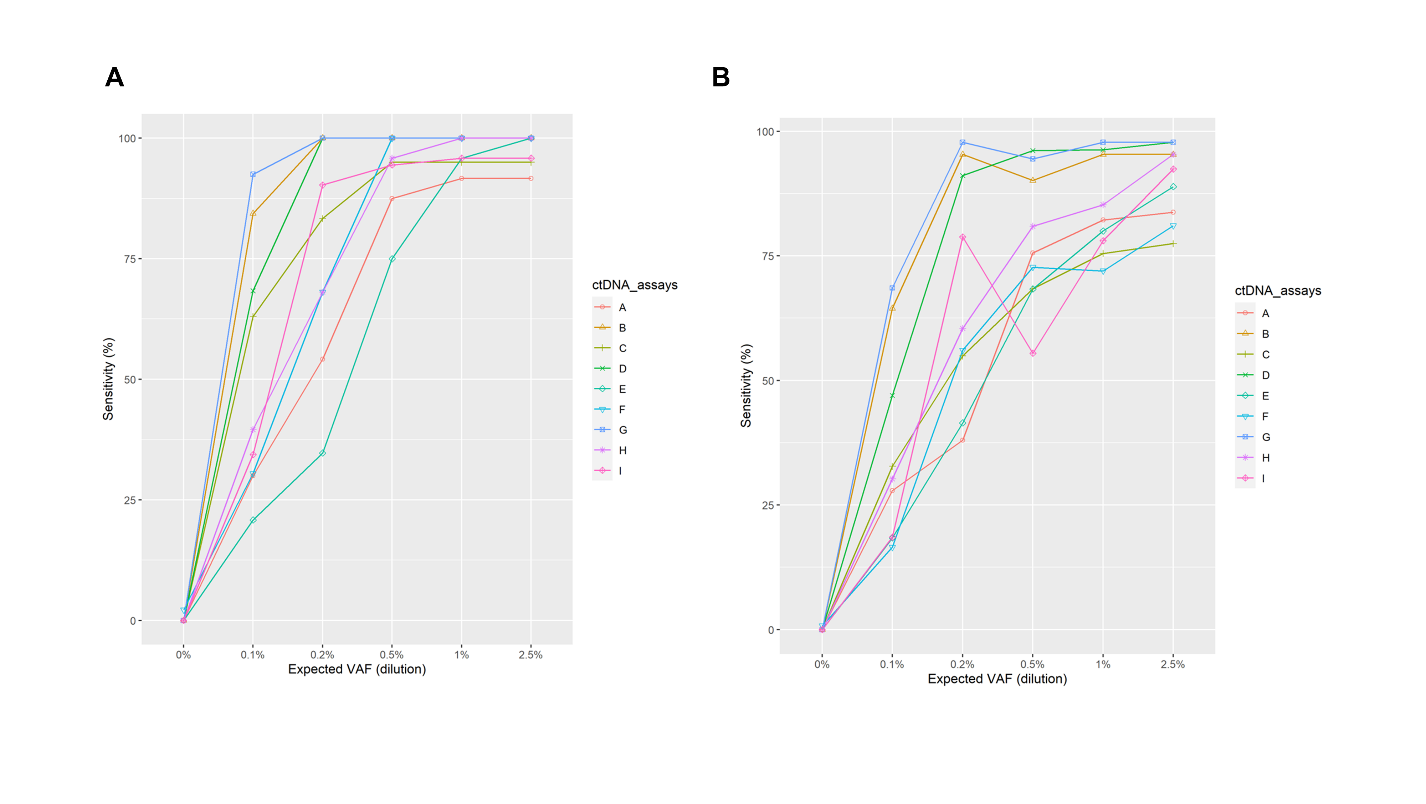
**

**Fig. S2 Comparison of assay sensitivity overall using VAF 2.5% as a reference.** Single nucleotide variants ≥20 ng (A) and overall (B). Related to Table 1. Similar patterns of sensitivity results among different assays were observed using the variants detected at 2.5% VAF as reference compared with the sensitivity results derived from using the overlapped variants in the panel as reference.

VAF, variant allele frequency
