# Supplementary material for: Analytical evaluation of circulating tumor DNA sequencing assays
Source: Sci Rep. 2024 Feb 29;14:4973. doi: 10.1038/s41598-024-54361-w (PMC10904763; doi:10.1038/s41598-024-54361-w)
Supplement: Supplementary file 4 — Supplementary Figure S3. [file 41598_2024_54361_MOESM4_ESM.docx]

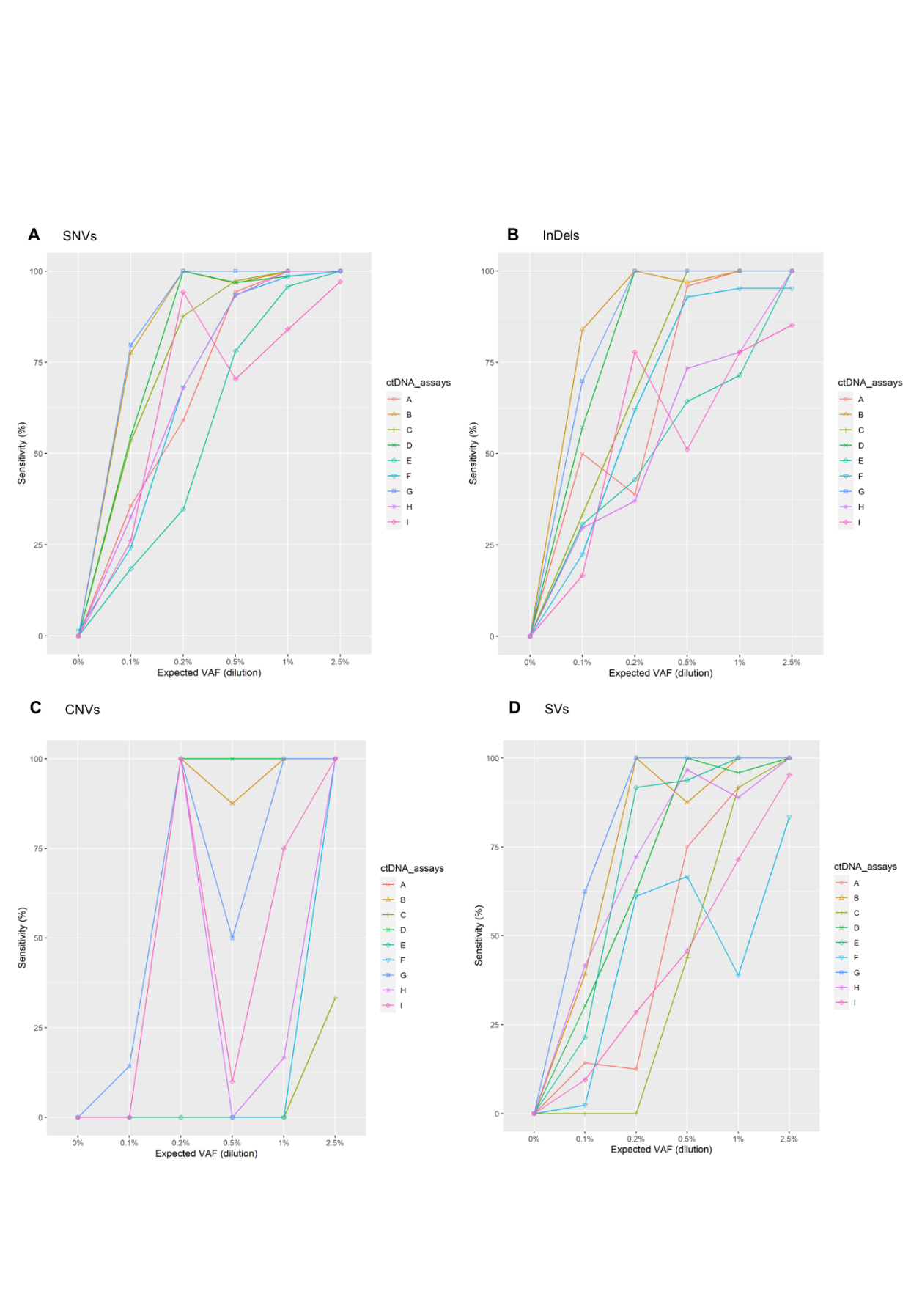


**Fig.** **S3 Sensitivity comparison according to variant type (A) SNVs, (B) InDels, (C) CNVs, and (D) SVs using VAF 2.5% as a reference.** Related to Fig 2. Similar patterns of sensitivity results among different assays with individual variant type were observed using the variants detected at 2.5% VAF as reference compared with the sensitivity results derived from using the overlapped variants in the panel as reference.

CNV, copy number variant; InDel, insertion or deletion variant; SNV, single nucleotide variant; SV, structural variant; VAF, variant allele frequency
